# Control Measures of Pathogenic Microorganisms and Shelf-Life Extension of Fresh-Cut Vegetables

**DOI:** 10.3390/foods10030655

**Published:** 2021-03-19

**Authors:** Jeong Yeon Lee, So Young Yang, Ki Sun Yoon

**Affiliations:** Department of Food and Nutrition, College of Human Ecology, Kyung Hee University, 26 Kyungheedae-ro, Dongdaemun-gu, Seoul 02447, Korea; ljyeon0902@naver.com (J.Y.L.); didthdud5253@naver.com (S.Y.Y.)

**Keywords:** fresh-cut vegetables, slightly acidic electrolyzed water (SAEW), ultrasounds (US), ultraviolet-C light-emitting diodes (UV-C LED), shelf life

## Abstract

We investigated the combined effect of using slightly acidic electrolyzed water (SAEW), ultrasounds (US), and ultraviolet-C light-emitting diodes (UV-C LED; 275 nm) for decreasing pathogenic *Escherichia coli* and *Staphylococcus aureus* (SEA) in fresh-cut vegetables, including carrots, celery, paprika, and cabbage. Survival of pathogenic *E. coli* and SEA and quality properties of fresh-cut vegetables at 5 and 15 °C for 7 days were also investigated. When combined treatment (SAEW + US + UV-C LED) was applied to fresh-cut vegetables for 3 min, its microbial reduction effect was significantly higher (0.97~2.17 log CFU/g) than a single treatment (*p* < 0.05). Overall, the reduction effect was more significant for SEA than for pathogenic *E. coli*. At 5 °C, SAEW + US and SAEW + US + UV-C LED treatments reduced populations of pathogenic *E. coli* and SEA in all vegetables. At 15 °C, SAEW + US + UV-C LED treatment inhibited the growth of both pathogens in carrot and celery and extended the shelf life of fresh-cut vegetables by preventing color changes in all vegetables. Although the effects of treatments varied depending on the characteristics of the vegetables and pathogens, UV-C LED can be suggested as a new hurdle technology in fresh-cut vegetable industry.

## 1. Introduction

Recently, purchase rates for fresh-cut vegetables such as ready-to-eat (RTE) food and meal kits have increased [1,2]. RTE fresh-cut vegetables generally refers to salads and stick-shaped and raw cut vegetables. They are generally consumed without washing or heating. They mainly include lettuce, cabbage, carrot, celery, cherry tomatoes, paprika, and so on [1,2,3,4]. However, due to non-thermal and minimal processing, contamination by pathogenic bacteria and viruses can be a risk factor during their production and distribution [5].

Foodborne illness outbreaks caused by pathogenic *Escherichia coli* and *Staphylococcus aureus* have been constantly occurring in Korea [6]. In the US and Europe, foodborne illness outbreaks at homes and restaurants due to consumption of fresh products contaminated by pathogenic *E. coli* O157:H7 and *E. coli* O104:H4 have been reported [7,8,9,10]. In addition, *S. aureus* and pathogenic *E. coli* have been consistently detected at high rates in agricultural products and RTE fresh-cut vegetables [11,12,13]. Recent research has also shown that some pathogens, including *L. monocytogenes*, *E. coli* O157:H7, *Salmonella* spp., *S. aureus*, *B. cereus*, and *C. jejuni*, have been isolated from various fruits and vegetables [14]. Therefore, appropriate measures to minimize contamination of foodborne pathogens and their effective control are required for RTE fresh-cut vegetables.

In general, sodium hypochlorite (NaClO) or a slightly acidic electrolyzed water (SAEW) is used in the washing and disinfection step as a critical control point (CCP) during the processing of fresh-cut vegetables [15,16]. Ultrasonic inactivation has been associated with damage to the cell wall and membrane of bacteria. However, it cannot be efficiently used in industrial applications because of its poor sterilization effect [17]. In recent studies, combined treatment with ultrasounds and SAEW has been applied, as it shows a synergistic bactericidal effect. Both ultrasounds and SAEW are known as safe, economical, and efficient methods [18,19]. Reduction effects of these disinfection treatments on pathogenic microorganisms in various vegetables have been studied [19,20,21].

Ultraviolet (UV) refers to light in the wavelength range of 100–400 nm. In this wavelength range, UV-C (200–280 nm) has the highest antibacterial activity [22,23]. Mercury-based UV-C lamps were frequently used for sterilization and disinfection in the past. However, they have been criticized due to mercury leak potential, short light life, and low energy efficiency [22]. As alternatives, semiconductor-based UV light-emitting diodes (LEDs) are attracting attention. LEDs can be developed in various wavelengths depending on the purpose. They also have excellent durability, energy efficiency, safety (not mercury), size, and long light life [24]. Ultraviolet-C light-emitting diodes’ (UV-C LED) treatment has been suggested as a new technology to inactivate pathogens [18,24].

UV-C LED treatment is more efficient than UV-A LED treatment (315–400 nm) for decreasing pathogenic microorganisms in various foods [25,26,27,28]. However, few studies have applied UV-C LED to vegetables products. In our preliminary study, *S. aureus* was detected qualitatively in five (5%) samples among 100 RTE fresh-cut vegetables and meal-kit products from online and offline markets. Thus, more efficient control measures than simple washing must be applied to RTE fresh-cut vegetables during processing for the safety of consumers.

Thus, objectives of this study were (1) to evaluate combined effects of using ultrasounds (US), slightly acidic electrolyzed water (SAEW), and ultraviolet-C light-emitting diodes (UV-C LED) on pathogenic *E. coli* and enterotoxin A-producing *S. aureus* before packaging step; (2) to extend shelf life of fresh-cut vegetables at retail markets.

## 2. Materials and Methods

### 2.1. Bacterial Strains

Pathogenic *Escherichia coli* including enteropathogenic *E. coli* (EPEC; NCCP 13715), enterotoxigenic *E. coli* (ETEC; NCCP 13717), and enterohemorrhagic *E. coli* O157:H7 (EHEC; NCTC 12079) producing verotoxins V1 and V2 were obtained from the Ministry of Food and Drug Safety (MFDS). Enterotoxin A-producing *Staphylococcus aureus* (SEA; ATCC 13565) was purchased from Korean Culture Center of Microorganisms (KCCM, Seoul, Korea). Each stock culture of pathogenic *E. coli* and *S. aureus* was maintained at −80 °C in tryptic soy broth (TSB, MBcell, Seoul, Korea) containing 20% glycerol. Frozen stocks were thawed for each experiment. After thawing, 10 μL of each strain was inoculated into 10 mL of sterile TSB and incubated at 36 °C for 24 h on a rotary shaker (VS-8480SP, Vision, Korea) at 140 rpm. Viable cell counts of pathogenic *E. coli* and *S. aureus* at the end of the incubation period ranged from 9.5 to 10.0 log CFU/g. For a cocktail strain of pathogenic *E. coli*, each bacterium culture was mixed in equal volumes. For spot-inoculation, 1 mL of starter culture at stationary phase was transferred into 9 mL of 0.1% sterilized peptone water (BD, Sparks, MD, USA) and then serially diluted before inoculation into samples. For dip-inoculation onto cabbage, 1 L (approximately 7 log CFU/g) of culture was prepared.

### 2.2. Preparation of Samples and Inoculation for Inactivation Study

According to our monitoring study, carrots, celery, paprika, and cabbage are the most common fresh-cut vegetables at a local market (Dongdemun-gu, Seoul, Korea). Each of these four raw vegetables was first washed in running tap water in order to remove soil and dust. After peeling off the outer skin, carrots were cut into stick-shaped pieces of 10 g each (length of 5~6 cm). After removing the outer two leaves and the core, cabbages were shredded to a thickness of 0.5 cm to prepare shredded cabbage samples (100 g each). Surfaces of carrots, celery, and paprika (10 g each) were inoculated with 100 μL of a three-strain cocktail of pathogenic *E. coli* and *S. aureus* producing enterotoxin A (SEA), respectively. Shredded cabbage (100 g) was dipped into 1 L of pathogenic *E. coli* and *S. aureus* suspensions (7 log CFU/g) and stirred for 2 min. Initial inoculation levels were approximately 5.5–6 log CFU/g. These inoculated samples were air-dried on a sterile stainless tray under a clean bench for 30 min.

### 2.3. Microbial Analysis

To investigate effect of each treatment for reducing the growth of pathogenic bacteria, viable cell count was measured using a spread plate culture method. Following disinfection treatment, 10 g of each treated sample was homogenized with 90 mL of sterile 0.1% peptone water using a stomacher (Interscience, Paris, France). The homogenate was serially diluted ten-fold with 0.1% peptone water and spread in duplicate onto selective media: eosin methylene blue agar (EMB, MBcell, Seoul, Korea) for pathogenic *E. coli* and Baird-Parker agar base (BPA, MBcell, Seoul, Korea) supplemented with egg yolk tellurite emulsion (MBcell, Seoul, Korea) for *S. aureus*. Tryptic soy agar (TSA, MBcell, Seoul, Korea), a non-selective medium, was used for both *E. coli* and *S. aureus*. Plates with selective and nonselective media were then incubated at 37 °C for 24~48 h. The number of colonies on each plate was then counted. Bacterial counts from duplicate plates were converted to log numbers.

### 2.4. Effect of Microbial Reduction with SAEW, US, and UV-C LED before Packaging

#### 2.4.1. Slightly Acidic Electrolyzed Water (SAEW) Treatment

Slightly acidic electrolyzed water (SAEW) at 30 ppm was produced using a SAEW generator (BC-120, Cosmic Round Korea Co., Seongnam, Korea) that basically consisted of a non-membrane electrolytic chamber with anode and cathode electrodes. The pH and available chlorine concentration of SAEW were measured with a pH meter (Orion-star pH-Bechtop, Thermo, USA) and a chlorine test paper (Toyo Roshi Kaisha, Ltd., Toyo, Japan), respectively, before SAEW was used to treat samples. The pH and available chlorine concentration of SAEW were 5.5 and 30 ppm, respectively.

#### 2.4.2. Ultrasound (US) Treatment

Ultrasound (US) treatment was performed using a sterile bench-top ultrasonic cleaner (POWERSONIC 620, Hwashin Tech Co., Ltd., Gyeonggi-do, Korea) at a fixed frequency of 40 kHz and an ultrasonic power of 700 W. After an ultrasound device (rectangular tank, 500 × 300 × 150 mm) was filled with 4 L of tap water, a stainless container (280 × 215 × 140 mm) was placed in this device and filled with SAEW prior to experiment. Ten samples (100 g) were immersed in SAEW.

#### 2.4.3. Combined Washing Treatment with SAEW and US

In order to investigate the optimal conditions of combined washing treatment with SAEW and US, the reduction effect of combined treatment was tested with carrot contaminated with EPEC alone under the following experimental conditions: time (0 and 3 min), the temperature of treatment solution (25 and 40 °C), and solution volume (10 and 20 times) with referring the previous works [21,29]. Carrots inoculated with EPEC were placed in a sterile stainless container filled with one or two liters of SAEW along with the ultrasound device. Reduction effects of EPEC in carrot under various conditions were compared as mentioned above. After treatments, samples were air-dried under a clean bench for 30 min. The most efficient reduction was observed for samples washed with SAEW for 3 min at ten times the sample volume in this preliminary work (Table 1). Thus, all fresh-cut vegetable samples (carrot, celery, paprika, and cabbage) were inoculated with pathogenic *E. coli* and *S. aureus* (SEA), respectively. They were treated in the same way.

#### 2.4.4. Treatment with Ultraviolet-C Light-Emitting Diodes (UV-C LED)

Five ultraviolet-C light-emitting diodes (UV-C LED, 275 nm) modules (Seoulviosys Co., Ltd., Gyeonggi-do, Korea) were connected onto bar-type electronic printed circuit boards (PCBs) of 45 cm in length. Direct current voltage (500 mA) from a power supply (TY-1006, Tae Young Electronics Co., Ltd., Gyeonggi-do, Korea) was applied to PCBs. UV-C LED treatment was performed in a chamber (500 × 400 × 520 mm) equipped with two PCBs. The UV-C radiation intensity of UV-C LEDs was measured with a UV light meter (UVC-254SD, Lutron Electronics Co., Inc., Coopersburg, PA, USA) calibrated for a range of 240~390 nm. It was measured to be 23 μW/cm^2^.

**Figure 1:**
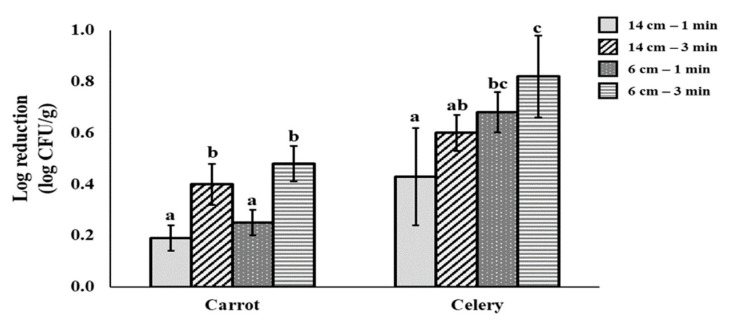
Reduction effect of UV-C LED irradiation (275 nm) for pathogenic *E. coli* (cocktail of EPEC, ETEC, and *E. coli* O157:H7) in fresh-cut carrot and celery at the various conditions. Distances of 6 and 14 cm, distance between the lights and samples; 1 and 3 min, irradiation time. The data were obtained from a non-selective media. ^a–c^ Values represent significant difference by Duncan’s multiple range test at *p* < 0.05.

To determine optimum irradiation conditions (processing time: 1 and 3 min; distance between sample and UV-C LED: 6 and 14 cm), effects of UV-C LED for reducing pathogenic bacteria were compared at each condition with two PCBs. The effect of microbial reduction was first tested for carrots and celeries contaminated with a three-strain cocktail of pathogenic *E. coli*. The superior performance of UV-C LED treatment was observed with an exposure time of 3 min and a distance between sample and UV-C LED source of 6 cm in this work (Figure 1). Then, prewashed and dried fresh-cut vegetables (carrot, celery, paprika, and cabbage, 10 g each) were inoculated with the three-strain cocktail of pathogenic *E. coli* and *S. aureus* (SEA), respectively, and then transferred to polyethylene terephthalate (PET) containers. They were placed in the chamber. The distance between each sample and the UV-C LED source was 6 cm. Samples were then irradiated with UV-C LED for 3 min. The UV-C dose at this condition was 4.14 mJ/cm^2^.

#### 2.4.5. Combined Treatment with SAEW, US, and UV-C LED

To evaluate the combined effect, combined treatment with SAEW, US, and UV-C LED was carried out in this study using conditions shown in Table 1. As a positive control, 100 ppm of sodium hypochlorite (NaClO; Hanson Hygiene Co., Chungcheongnam-do, Korea) solution was used. This solution was prepared by diluting 4% NaClO solution using distilled water.

### 2.5. Quality Evaluation for Treated Vegetables during Storage at 5 and 15 °C after Packaging

To evaluate qualities of fresh-cut vegetables, carrot, celery, paprika, and cabbage were treated with SAEW + US or SAEW + US + UV-C LED irradiation. Treated samples were packaged with PET containers (Modenpack, Seoul, Korea) and then stored at 5 °C (storage temperature) and 15 °C (abused temperature) for 7 days.

#### 2.5.1. Survival of Pathogenic E. coli and S. aureus on Fresh-Cut Vegetables after Combined Treatment

For fresh-cut vegetables inoculated with pathogenic *E. coli* and *S. aureus*, survival rates of these pathogens after treatments during storage were investigated and compared with those in the control group without any treatment. This experiment was repeated twice at different time.

#### 2.5.2. Effects of Combined Treatments on Quality Parameters of Fresh-Cut Vegetables

To investigate effects of applied treatments on physical qualities of fresh-cut vegetables, moisture loss, instrumental color, and appearance observation were measured. Moisture loss was determined by taking the difference between the initial weight of treated fresh-cut vegetable and that obtained one at the end of each storage time using the following formula:

(1) (%)Moisture loss=(initial mass−final mass)÷initial mass×100

The color of each sample was measured using a colorimeter (Minolta CR-400, Osaka, Japan) every day for 7 days. The instrument was standardized against a white standardization plate before each measurement. All measurements were performed in triplicate. Color was described as coordinates: lightness (L*), redness (a*, ±red-green), and yellowness (b*, ±yellow-blue).

### 2.6. Statistical Analysis

Experiments for US, SAEW, and UV-C LED treatments were repeated twice with three replicates per treatment. All statistical analyses were conducted with the Statistical Analysis System SAS V 9.4 (SAS Institute Inc., Cary, NC, USA). The significance of differences among or between samples was determined by one-way ANOVA followed by Duncan’s test for multiple range tests or *t*-test at *p* < 0.05.

## 3. Results and Discussion

### 3.1. Effect of Microbial Reduction with SAEW, US, and UV-C LED before Packaging

#### 3.1.1. Effects of Volume and Temperature of Combined Treatment with SAEW and US

First of all, reduction effects of combined treatments on EPEC in carrots were compared under various conditions (temperature 25 and 40 °C and volume of treatment solution 10 and 20 times) as described previously [21,29]. Microbial reduction effects of combined treatments were not significantly different regardless of temperature or volume of washing solution (*p* < 0.05) (Table 2). Previous studies have reported that higher temperature of treatment solution (25~60 °C) is more effective for reducing pathogenic bacteria in fresh-cut carrot, potato, and bell pepper [21,29,30]. However, potatoes washed at 60 °C with US treatment have shown color changes [29]. Other studies have reported that dipping in electrolyzed water for 3 min has the best sanitizing effect [31,32,33]. The present study also showed that washing for 3 min with SAEW + US at 10 times the sample volume at 25 °C was the most effective treatment condition to reduce the population of EPEC in fresh-cut carrot. Ultrasonic inactivation has been associated with damage to the cell wall and membrane of bacteria. Ultrasound can enhance the inactivation of microorganisms in chemical solutions such as SAEW [20,34,35].

#### 3.1.2. Effects of Time and Distance between Sample and UV-C LED source

To determine optimum irradiation conditions such as application time (1 and 3 min) and distance between a sample and UV-C LED (6 and 14 cm), reduction effects of UV-C LED were compared with pathogenic *E. coli* cocktail strains in carrot and celery at different conditions. The highest reduction was observed for both carrot and celery after UV-C irradiation for 3 min with a distance of 6 cm (Figure 1). Thus, this was confirmed as the optimal condition under the present UV-C irradiation dose (4.14 mJ/cm^2^). Reduction levels of *E. coli* O157:H7, *S.* Typhimurium, and *L. monocytogenes* in culture media under UV-C (266~279 nm) dose of 1.67~3 mJ/cm^2^ have been reported to be 3~6 log CFU/g in previous works [36].

#### 3.1.3. Effect of Microbial Reduction in Combined Treatments with SAEW, US, and UV-C LED before Packaging

Finally, synergistic effects of combined treatments were evaluated to reduce pathogenic *E. coli* and *S. aureus* (SEA) in carrot, celery, paprika, and cabbage at the optimal condition of the present study, as shown in Table 3. Results revealed that the combined treatment of 30 ppm SAEW + US (0.61~1.84 log CFU/g reduction) had a greater washing effect than a single treatment by 30 ppm SAEW (0.49~1.39 log CFU/g reduction) for both pathogenic *E. coli* and *S. aureus* (SEA) in all vegetables. Additionally, application of UV-C LED irradiation as a pre-packaging step after washing with SAEW or SAEW + US increased the reduction effect compared to UV-C LED irradiation only. Combined treatment of 30 ppm SAEW + US + UV-C LED show higher reduction effect than 100 ppm NaClO solution alone, indicating that UV-C LED could be used as a new hurdle technology in the industry of fresh-cut vegetables. Regardless of the kind of pathogen, the highest reduction was observed with SAEW + US + UV-C LED (0.97~2.17 log CFU/g) for all four vegetable samples.

Combined washing with SAEW and US has been reported to be effective for microbial decontamination and shelf life extension of various vegetables [21,37,38,39] and kashk [40]. Reduction levels of *E. coli* O157:H7 have been observed to be 1.3~3.3 log CFU/g in Chinese cabbage, lettuce, sesame leaf, spinach, and kale after washing with SAEW and US [37,39]. Combined washing with SAEW and US for kashk has also shown a reduction effect of 1.7 log CFU/g for *E. coli* O157:H7 and 1.9 log CFU/g for *S. aureus* [40]. Maximum reduction effect of washing with SAEW and US for *B. cereus* biofilms has been reported to be 4 log CFU/g on leafy green vegetables [38]. In our study, a combined washing treatment with 30 ppm SAEW and US showed similar reduction levels as those shown in other studies.

Recent studies have reported reduction effects of UV-C LED irradiation (254~280 nm wavelength) on apple juice and apple [26,27]. Jiang et al. [28] have also investigated the effect of combined treatment with SAEW and UV-C LED for inactivating *Salmonella* Typhimurium and *E. coli* O157:H7 on coriander. Their study also used 4 UV-C doses of 8~432 mJ/cm^2^ along with SAEW washing. The reduction level of pathogens was 1.50~2.42 log CFU/g, similar to our results with UV-C LED irradiation (275 nm) at a dose of 4.14 mJ/cm^2^.

Green et al. [41] have compared effects for inactivating *E. coli* O157:H7 surrogate using a low-pressure mercury (LPM) lamp (253.7 nm) and UV-C LED (275 nm) at UV-C dose 0~20 mJ/cm^2^. UV-C LED irradiation for the same period of time resulted in a larger log reduction (~5 log CFU/g) than the LPM lamp, since a warm-up time was not required for UV-C LED irradiation compared to the LPM lamp. Reduction levels of *S. aureus* on lettuce and strawberry were also observed to be 1.21 and 0.5 log CFU/g, respectively, after applying UV-C LPM lamp irradiation (254 nm) for 45 min (540 mJ/cm^2^) [42]. These results indicate that UV-C LED could be efficiently used with a shorter time and a less consumption of energy than UV-C LPM lamp.

#### 3.1.4. Effect of the Kind of Pathogen and Vegetables on Microbial Reduction with SAEW, US, and UV-C LED

In the present study, a more effective reduction was observed for SEA (0.50~2.17 log CFU/g) than for pathogenic *E. coli* (0.41~1.59 log CFU/g) in all vegetables except cabbage (Table 3). Generally, Gram-positive bacteria are recognized as more resistant than Gram-negative bacteria, since they have thicker cell walls that can protect against physical and chemical treatments [34,43]. Pathogenic *E. coli* (0.30~1.27 log CFU/g) showed greater reduction than SEA (0.34~0.97 log CFU/g) in cabbage of this study. No significant difference in inactivation level between Gram-positive and Gram-negative bacteria after treatment with ultrasound has been reported [44]. On the other hand, Crook et al. [45] have reported that *S. aureus* is the most sensitive bacterium among seven pathogens (*L. monocytogenes*, *E. coli*, *Serratia marcescens*, *Salmonella* Senftenberg, *Yersinia enterocolitica*, *Aeromonas hydrophila*, and *S. aureus*) to UV-C irradiation.

**Figure 2:**
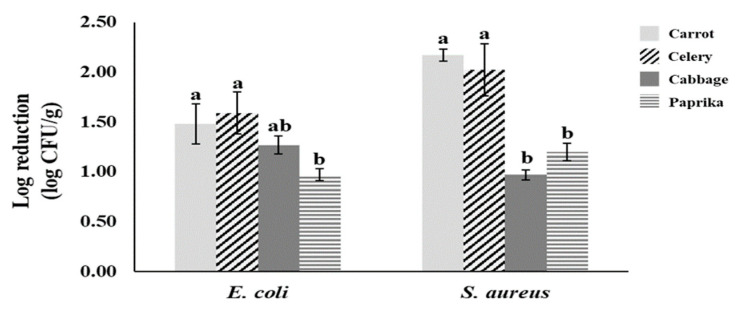
Comparison for reduction effect of combined treatment with SAEW + US + UV-C LED on pathogenic *E coli* (cocktail of EPEC, ETEC, and *E. coli* O157:H7) and *S. aureus* (SEA) in various vegetables. The data were obtained from a non-selective media. ^a,b^ Values within each treatment represent different by Duncan’s multiple tests at *p* < 0.05.

Regarding the kind of vegetable, all treatments were more effective for pathogens in carrot and celery than in paprika and cabbage. When combined treatment of US + SAEW + UV-C LED was applied, the highest reduction levels for pathogenic *E. coli* and SEA were measured to be 1.48 and 2.17 log CFU/g, respectively, in carrot, and 1.59 and 2.02 log CFU/g, respectively, in celery (Table 3, Figure 2). A significantly greater reduction with SAEW + US was observed for pathogenic *E. coli* and SEA in carrot, while a more effective UV-C LED treatment was observed for both pathogens in celery (*p* < 0.05). Additional UV-C LED irradiation enhanced decontamination effects for carrot and celery. However, these reinforced effects were not significantly different between carrot and celery (*p* > 0.05). Surface characteristic of fresh produce was an important factor affecting inactivation effects of physicochemical treatments such as SAEW, US, and UV-C LED in most studies. Inactivation rates were higher for less hydrophobic fruits with smoother surfaces (apple, pears, and cherry tomato) than for those with rougher surfaces (cantaloupe, strawberry, and raspberry) [20,46].

The surface of celery itself is smoother than that of carrot. However, due to a lot of regular groove shapes, a more significant reduction effect of SAEW + US was observed for carrots than for celeries during this study’s washing step. However, a better disinfection effect was observed for celeries than for carrots with UV-C LED irradiation. In the present study, a relatively lower microbial reduction effect was observed for paprika and cabbage.

Moreover, a similar reduction effect in pathogenic *E. coli* and SEA was noticed in cabbage and paprika.

Since the effect of disinfection treatment was affected by various factors such as surface properties of vegetables, adherence characteristics of pathogen on the surface of different vegetables, additional hurdle technology might be needed during processing and distribution of various kinds of fresh-cut vegetables.

### 3.2. Quality Evaluation of Treated Vegetables during Storage at 5 and 15 °C

#### 3.2.1. Survival of Pathogenic E. coli and S. aureus on Fresh-Cut Vegetables after Combined Treatment

**Figure 3:**
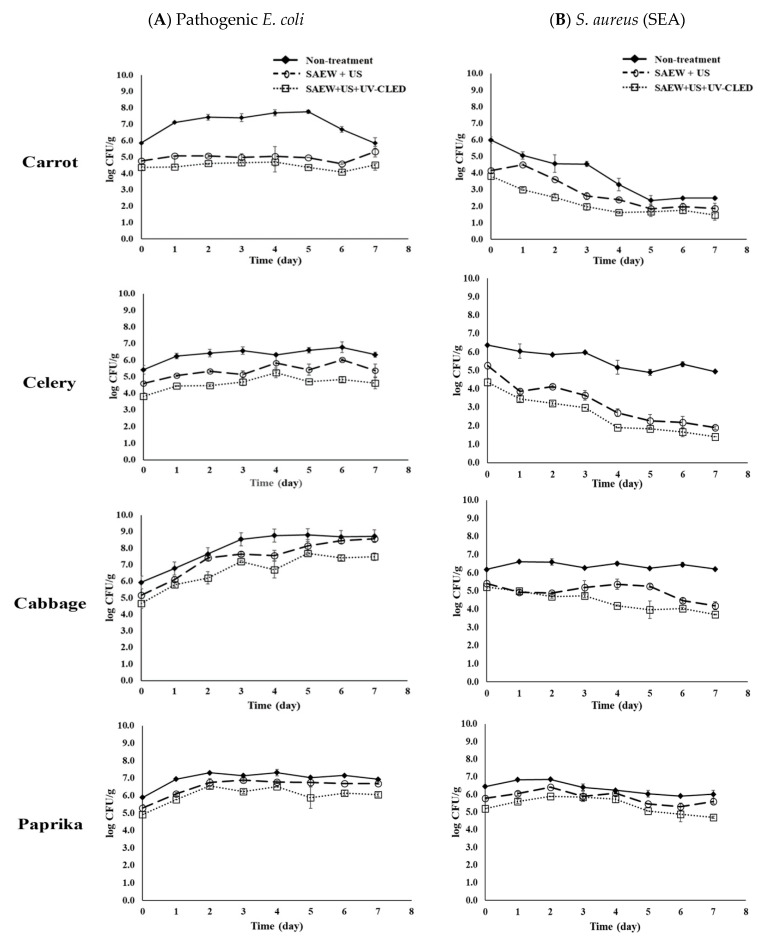
Effect of combined treatment on the survival of (**A**) pathogenic *E. coli* (cocktail of EPEC, ETEC, and *E. coli* O157:H7) and (**B**) *S. aureus* (SEA) in fresh-cut carrot, celery, cabbage and paprika stored for 7 days at 15 °C. Non-treatment, no extra wash with a sanitizer; SAEW, slightly acidic electrolyzed water; US, ultrasounds; UV-C LED, ultraviolet-C light-emitting diodes (275 nm); the data were obtained from a selective media.

After combined treatment with SAEW + US + UV-C LED, survival characteristics of pathogenic *E. coli* and enterotoxin A-producing *S. aureus* (SEA) inoculated on fresh-cut carrot, celery, cabbage, and paprika were investigated after storage at 5 °C (storage temperature) and 15 °C (abused temperature) for 7 days. At 5 °C, the combined treatment decreased both pathogens’ populations to the lowest level (1.5 logs CFU/g) in all vegetables (data not shown). At 15 °C, the growth of pathogenic *E. coli* was inhibited in only carrot and celery treated with SAEW + US or SAEW + US + UV-C LED during storage (Figure 3A). The population of *E. coli* treated with SAEW + US + UV-C LED was increased up to 7.7 log CFU/g in cabbage, which was lower than the population in the non-treated sample (8.8 log CFU/g). The growth of *E. coli* was not also inhibited in paprika treated with SAEW + US or SAEW + US + UV-C LED.

On the other hand, the combined treatment decreased SEA populations to the lowest level (1.5 log CFU/g) in carrots and celeries after storage at 15 °C for 7 days (Figure 3B). Similar results were found after storage at 5 °C. The populations of SEA decreased in cabbage treated with SAEW + US or SAEW + US + UV-C LED, while SEA population was not changed in the non-treated cabbage during 7 days of storage at 15 °C. On the other hand, the populations of SEA were not changed in paprika during 7 days of storage at 15 °C, regardless of treatment, indicating that SEA in paprika was the least affected by SAEW + US or SAEW + US + UV-C LED. Overall, the growth control was more effective for SEA in all vegetables than for pathogenic *E. coli* based on reduction effects of treatments (Table 3, Figure 3). A more rapid reduction in SEA in carrots and celeries than in cabbage and paprika during storage at 15 °C was noticed.

Previous studies have reported the survival of foodborne pathogens in vegetables after different treatments [19,32]. Total bacterium counts on lettuce and button mushroom applied combined treatment with SAEW and US are lower than those without treatments after 6–8 days of storage at 4 and 10 °C [19,32]. Jiang et al. [28] have also studied the reduction effect of combined treatment with SAEW and UV-LED on *Salmonella* Typhimurium and *E. coli* O157:H7 in coriander after 6 days of storage at 4 °C. Among tested samples, SAEW + UV-LED-treated samples showed the lowest levels of *Salmonella* Typhimurium and *E. coli* O157:H7. Combined treatment of SAEW, US, and UV-C LED show synergistic effects due to the impact of chlorine compounds, cavitation, and ultraviolet-C, causing structural damage of cell membrane efficiently [17,26,47,48]. A synergistic effect was also observed for pathogenic bacteria on vegetables treated with SAEW + US + UV-C LED in the present study, indicating that such combined technology might be effective in extending the shelf life of fresh-cut vegetables. The most effective method of elimination Shiga toxin (verotoxin)-producing *E coli* (STEC) from foods is to introduce a bactericidal treatment, such as heating or gamma irradiation [49,50]. The present results show that UV-C LED treatment with washing can replace the gamma irradiation in the fresh-cut vegetable industry. However, the retail market’s storage temperature must not be abused, since the growth of pathogenic *E. coli* was not prevented by such treatment at 15 °C.

Overall, the growth of pathogens in carrot and celery was controlled better than in cabbage and paprika at 5 and 15 °C. Antimicrobial activities of water-soluble polysaccharides in carrot and sedanolide (aroma component) in celery might explain such results [51,52]. Consequently, it can be concluded that combined treatment may extend the shelf life of fresh-cut vegetables, although the microbial reduction effect may vary depending on the characteristics of vegetables.

#### 3.2.2. Effects of Combined Treatments on Quality Parameters of Fresh-Cut Vegetables

**Figure 4:**
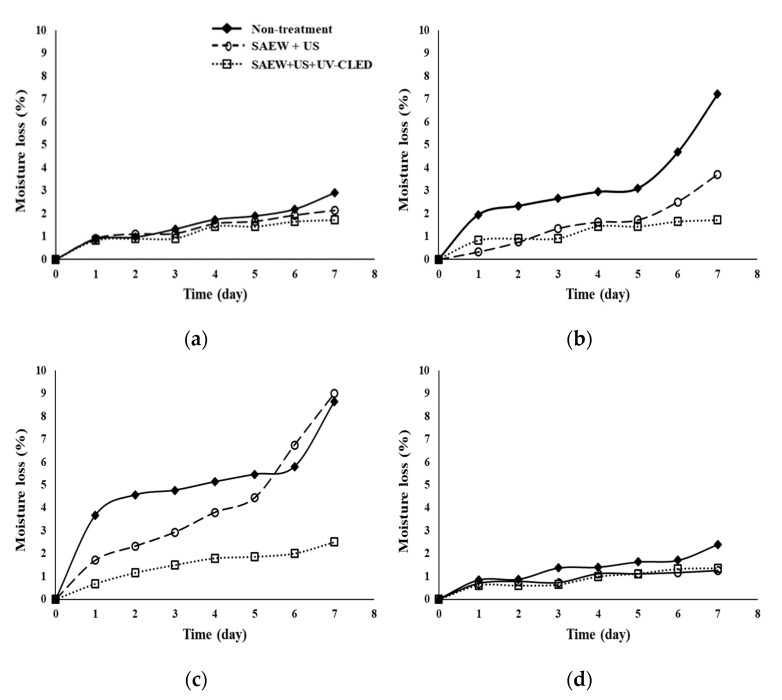
Effect of combined treatment on the moisture loss in various fresh-cut vegetables stored for 7 days at 5 °C. (**a**) Carrot, (**b**) celery, (**c**) cabbage, (**d**) paprika; non-treatment, no extra wash with a sanitizer; SAEW, slightly acidic electrolyzed water; US, ultrasounds; UV-C LED, ultraviolet-C light-emitting diodes (275 nm); non-treatment, no extra wash with a sanitizer.

**Figure 5:**
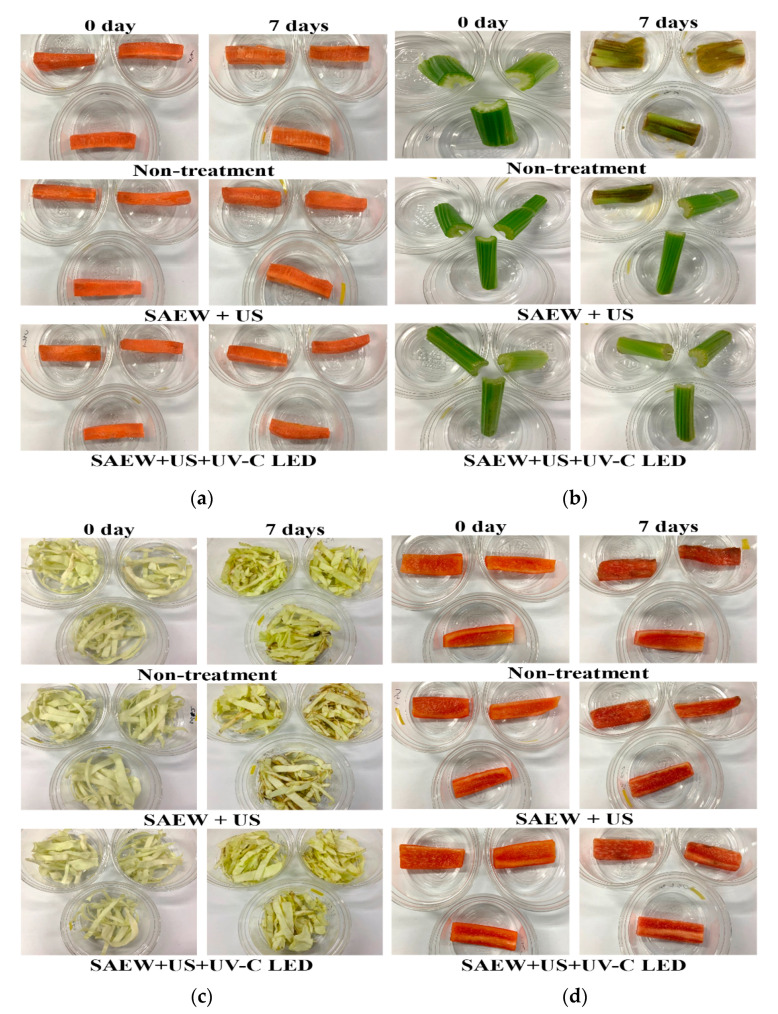
Effect of combined treatments on visible qualities in various fresh-cut vegetables after storage for 7 days at 15 °C. (**a**) Carrot, (**b**) celery, (**c**) cabbage, (**d**) paprika; non-treatment, no extra wash with a sanitizer; SAEW, slightly acidic electrolyzed water; US, ultrasounds; UV-C LED, ultraviolet-C light-emitting diodes (275 nm).

To evaluate the effects of various treatments on quality parameters of fresh-cut carrot, celery, cabbage, and paprika, moisture loss (Figure 4), appearance (Figure 5), and instrumental color (Table 4) were measured during storage at 5 and 15 °C for 7 days. Figure 4 shows changes in moisture in samples treated with SAEW + US or SAEW + US + UV-C LED and stored for 7 days at 5 °C compared to non-treated samples.

The lowest moisture loss with SAEW + US + UV-C LED treatment was noticed for celery (1.4%) and cabbage (2.5%) after storage for 7 days. Such moisture loss was lower than that with SAEW + US or non-treated samples (3.7~9.0%). However, changes in moisture in carrot (Figure 4a) and paprika (Figure 4d) were observed to have similar levels at all conditions. A similar tendency of moisture loss was noticed at 15 °C (data not shown). Generally, a loss of moisture greater than 5% would cause a reduction in the retail value of vegetables and fruits [53]. The threshold level of moisture loss is 10% for ending the shelf life of fresh produce [54]. Regardless of treatment, less than 5% of moisture loss was observed for carrot and paprika. Less than 5% of moisture loss was also observed for celery and cabbage treated with SAEW + US or SAEW + US + UV-C LED in the present study. Other studies have also reported that moisture losses in tomatoes treated with the US and UV-C [55] and yellow bell pepper treated with UV-C irradiation [56] are lower than those in non-treated samples. Treatments with the US and various sanitization agents did not affect purple cabbage moisture during storage [57]. It is also reported that the combination of SAEW and ultrasound had no significant effect on the contents of total soluble solids, vitamin C, or total titratable acidity in both strawberries and cherry tomatoes [20]. Total phenol and ascorbic acid contents of UV-C-treated yellow bell pepper remained constant during refrigerated storage [56]. These results showed that UV-C is an effective non-chemical treatment to maintain the qualities of fruits and vegetables.

Appearance changes in fresh-cut vegetables stored at 15 °C are shown in Figure 5. After 7 days of storage, conspicuous appearance changes in non-treated celery, cabbage, and paprika were identified at an abused temperature of 15 °C compared to samples treated with SAEW + US + UV-C LED. However, appearance changes were not noticed for non-treated samples and samples treated with a combination of methods after storage at 5 °C for 7 days (data not shown). Overall, significant changes in values of L*, a*, and b* were not observed for samples treated with SAEW + US + UV-C LED after 7 days of storage at 15 °C compared to SAEW + US or non-treated samples (*p* > 0.05) (Table 4). Based on visual inspection for sliced button mushrooms, application of SAEW and US treatments delayed browning after 8 days of storage at 5 °C [19]. Other previous works [56,57,58] have also reported that L*, a*, and b* values were not affected for purple cabbage, tomato, and yellow bell pepper treated with the US, chemical sanitizer, and UV-C technologies during storage at 8~12 °C. The present study also proved that combined treatment of SAEW + US + UV-C LED prevented the color change in fresh-cut vegetables and could be applied to maintain the quality and safety of fresh-cut vegetables.

## 4. Conclusions

In the present study, combined treatment with 30 ppm SAEW + US + UV-C LED before packaging was more effective in reducing microbial contamination than every single treatment. Combined treatment with SAEW, US, and UV-C LED further reduced populations of pathogenic *E. coli* and enterotoxin A-producing *S. aureus* (SEA) in fresh-cut vegetables. Combined treatments (SAEW + US or SAEW + US + UV-C LED) reduced populations of pathogenic *E. coli* and SEA to the lowest levels in all vegetables after storage at 5 °C for 7 days. The growth of pathogenic *E. coli* and SEA was more effectively inhibited in carrot and celery than in cabbage and paprika treated with SAEW + US + UV-C LED during storage at an abused temperature of 15 °C. Overall, the growth control by combined treatment was more effective for SEA than for pathogenic *E. coli*. It was also noticed that combined treatment with SAEW + US + UV-C LED prevented moisture loss, color, and appearance change during storage at 5 and 15 °C. These results indicate that combined treatment of SAEW, US, and UV-C LED can be applied as a useful technology to extend the shelf life of fresh-cut vegetables. However, its effect may vary depending on the characteristics of vegetables. Especially, UV-C LED can be suggested as a new hurdle technology in the fresh-cut vegetable industry.

## Figures and Tables

**Table 1:** Application conditions of single or combined treatments with SAEW, US, and UV-C LED applied for fresh-cut vegetables.

Treatments	Application Conditions
NaClO	Washing ^1^ with NaClO
SAEW	Washing with SAEW
SAEW + US	Washing with SAEW and US
UV-C LED	UV-C LED irradiation ^2^
SAEW + UV-C LED	Washing with SAEW → UV-C LED irradiation
SAEW + US + UV-C LED	Washing with SAEW and US → UV-C LED irradiation

^1^ washing for 3 min with SAEW + US at 10 times volume of the sample at 25 °C. ^2^ Irradiation for 3 min and 6 cm distance between sample and UV-C LED source. NaClO, sodium hypochlorite solution (control, 100 ppm); SAEW, slightly acidic electrolyzed water (30 ppm); US, ultrasounds; UV-C LED, ultraviolet-C light-emitting diodes (275 nm).

**Table 2:** Effects of volume and temperature on pathogenic *E. coli* (EPEC) population in fresh-cut carrot by combined treatment with SAEW and US for 3 min.

Volume of SAEW ^1^	Log Reduction (log CFU/g)	
	25 °C ^2^	40 °C
10 times	1.15 ± 0.15 ^3^	1.04 ± 0.08
20 times	1.02 ± 0.06	* 1.31 ± 0.06 **

^1^ The volume of SAEW was a multiple of the sample volume.^2^ The temperature of SAEW. ^3^ The data were obtained from a non-selective media. * Significant difference between the volume of SAEW was observed at each temperature (*p* < 0.05). ** Significant difference between the temperature of SAEW was observed at each volume (*p* < 0.05).

**Table 3:** Reduction populations of pathogenic *E. coli* (cocktail of EPEC, ETEC, and *E. coli* O157:H7) and *S. aureus* (SEA) in various fresh-cut vegetables by disinfection treatments.

Treatments	Log Reduction (log CFU/g)							
	Carrot		Celery		Cabbage		Paprika	
	*E. coli*	*S. aureus*	*E. coli*	*S. aureus*	*E. coli*	*S. aureus*	*E. coli*	*S. aureus*
NaClO	* 1.30 ± 0.19 ^AB^	0.93 ± 0.18 ^D^	1.34 ± 0.52 ^AB^	1.70 ± 0.38 ^AB^	0.90 ± 0.03 ^C^	0.81 ± 0.09 ^B^	* 1.15 ± 0.04 ^A^	0.58 ± 0.18 ^BC^
SAEW	0.67 ± 0.13 ^C^	* 1.39 ± 0.27 ^C^	0.58 ± 0.12 ^C^	0.76 ± 0.18 ^D^	* 0.68 ± 0.05 ^D^	0.49 ± 0.04 ^C^	0.54 ± 0.08 ^D^	0.50 ± 0.02 ^C^
SAEW + US	1.09 ± 0.22 ^B^	* 1.84 ± 0.24 ^B^	0.83 ± 0.12 ^BC^	1.11 ± 0.47 ^BC^	0.77 ± 0.09 ^D^	0.77 ± 0.09 ^B^	0.61 ± 0.16 ^D^	0.67 ± 0.08 ^BC^
UV-C LED	0.48 ± 0.07 ^C^	* 0.76 ± 0.11 ^D^	0.82 ± 0.13 ^BC^	* 1.37 ± 0.31 ^AC^	0.30 ± 0.08 ^E^	0.34 ± 0.21 ^D^	0.41 ± 0.08 ^E^	* 0.57 ± 0.06 ^BC^
SAEW + UV-C LED	1.10 ± 0.17 ^B^	* 1.54 ± 0.27 ^C^	1.04 ± 0.27 ^BC^	1.60 ± 0.48 ^ABC^	* 1.13 ± 0.14 ^B^	0.75 ± 0.09 ^B^	0.85 ± 0.10 ^C^	0.74 ± 0.12 ^B^
SAEW + US + UV-C LED	1.48 ± 0.20 ^A^	* 2.17 ± 0.27 ^A^	1.59 ± 0.21 ^A^	2.02 ± 0.44 ^A^	* 1.27 ± 0.09 ^A^	0.97 ± 0.05 ^A^	0.97 ± 0.06 ^B^	1.20 ± 0.26 ^A^

NaClO, sodium hypochlorite solution (control, 100 ppm); SAEW, slightly acidic electrolyzed water (30 ppm); US, ultrasounds; UV-C LED, ultraviolet-C light-emitting diodes (275 nm). The data were obtained from a non-selective media. ^A–E^ Within the same column, values not followed by the same uppercase letter are significantly different (*p* < 0.05). * Significant difference between *E. coli* and *S. aureus* was observed at each vegetable (*p* < 0.05).

**Table 4:** Effect of combined treatments on hunter color values in fresh-cut vegetables after storage for 7 days at 15 °C.

Samples		L*		a*		b*	
		0 Day	7 Days	0 Day	7 Days	0 Day	7 Days
Carrot	Non-treatment	55.46 ± 1.30 ^1^	* 57.02 ± 0.53	32.79 ± 0.26	* 25.91 ± 1.06	29.73 ± 1.59	* 24.44 ± 1.37
	SAEW + US	50.24 ± 0.59	* 53.96 ± 1.90	31.57 ± 1.15	* 27.49 ± 0.89	29.11 ± 0.47	* 26.04 ± 2.30
	SAEW + US + UV-C LED	50.66 ± 0.93	51.94 ± 1.79	30.38 ± 0.14	30.39 ± 0.33	29.55 ± 0.41	* 27.29 ± 0.17
Celery	Non-treatment	50.68 ± 1.12	* 36.48 ± 0.16	−13.93 ± 0.15	* −3.20 ± 1.13	21.58 ± 1.16	* 15.22 ± 0.08
	SAEW + US	44.78 ± 1.20	42.54 ± 2.18	−13.13 ± 0.32	* −7.00 ± 0.25	18.75 ± 0.42	18.16 ± 1.48
	SAEW + US + UV-C LED	48.43 ± 2.84	50.44 ± 2.99	−14.01 ± 0.26	−12.96 ± 1.77	20.67 ± 1.13	21.30 ± 1.42
Cabbage	Non-treatment	80.88 ± 2.45	* 64.03 ± 0.47	−3.30 ± 1.20	* −6.60 ± 1.86	9.85 ± 0.68	* 17.81 ± 0.90
	SAEW + US	79.56 ± 1.94	* 75.07 ± 0.28	−3.72 ± 0.25	* −5.69 ± 0.51	11.08 ± 0.04	* 16.97 ± 0.50
	SAEW + US + UV-C LED	79.12 ± 1.72	77.38 ± 2.23	−3.21 ± 0.46	−3.55 ± 0.30	10.24 ± 2.41	* 14.97 ± 0.53
Paprika	Non-treatment	31.55 ± 0.71	* 27.37 ± 1.54	14.48 ± 0.21	* 11.97 ± 0.16	12.00 ± 0.65	* 10.53 ± 0.50
	SAEW + US	32.81 ± 0.08	* 28.21 ± 1.03	14.82 ± 0.42	14.40 ± 0.59	11.64 ± 0.48	* 10.67 ± 0.64
	SAEW + US + UV-C LED	31.10 ± 1.41	30.29 ± 0.88	15.18 ± 0.20	15.52 ± 0.42	10.76 ± 0.09	10.23 ± 0.46

Non-treatment, no extra wash with a sanitizer, SAEW, slightly acidic electrolyzed water; US, ultrasounds; UV-C LED, ultraviolet-C light-emitting diodes (275 nm). ^1^ The average value (*n* = 6) ± standard deviation of two experiments. During each experiment at least three samples of each vegetable were taken. Each value was measured for at least five points in each vegetable.* Significant difference between stored vegetable samples for 0 day and 7 days was observed at each condition (*p* < 0.05).

## Data Availability

We did not report any additional data for this study.
